# Case Report: Immune-directed post-transplant maintenance with low-dose gemtuzumab ozogamicin and stepwise donor lymphocyte infusion in acute myeloid leukemia with t(1;3)(p36.3;q21)

**DOI:** 10.3389/fimmu.2026.1888993

**Published:** 2026-08-05

**Authors:** Fumihiko Ouchi, Yuho Najima, Kyoko Haraguchi, Daichi Sadato, Chizuko Hirama, Kaoru Morita, Takashi Toya, Hiroaki Shimizu, Tomomi Toubai, Hironori Harada, Yuka Harada, Noriko Doki

**Affiliations:** 1 Hematology Division, Tokyo Metropolitan Komagome Hospital, Tokyo, Japan; 2 Division of Transfusion and Cell Therapy, Tokyo Metropolitan Komagome Hospital, Tokyo, Japan; 3 Clinical Research and Trials Center, Tokyo Metropolitan Komagome Hospital, Tokyo, Japan; 4 Laboratoty of Oncology, Tokyo University of Pharmacy & Life Sciences, Tokyo, Japan

**Keywords:** acute myeloid leukemia, allogeneic hematopoietic stem cell transplantation, donor lymphocyte infusion, gemtuzumab ozogamicin, graft-versus-leukemia effect, post-transplant maintenance, t(1;3)(p36.3;q21)

## Abstract

Relapse after allogeneic hematopoietic stem cell transplantation (allo-HSCT) is the leading cause of treatment failure in acute myeloid leukemia (AML), particularly in genetically adverse subtypes. Although prophylactic donor lymphocyte infusion (DLI) may augment graft-versus-leukemia effects, its stand-alone efficacy is suboptimal, necessitating rational combination approaches. Limited case reports have suggested potential clinical benefits of gemtuzumab ozogamicin (GO) in combination with DLI as salvage therapy for post-transplant relapse; however, its role in prophylactic maintenance has not been determined. We report a 29-year-old woman with chemo-refractory AML harboring t(1;3)(p36.3;q21) who relapsed approximately 3 months after her first allo-HSCT. She underwent a second haploidentical allo-HSCT without achieving remission beforehand. Given the extremely high risk of relapse, she received immune-directed maintenance with low-dose GO (3 mg/m^2^ on days 98 and 140 post-transplant) followed by dose-escalated DLI (six monthly infusions starting on day 374). Although the patient developed chronic graft-versus-host disease, including pulmonary involvement (NIH lung score 2), it remained clinically manageable. She maintained molecular complete remission for more than 6 years. Our case suggests that sequential low-dose GO followed by stepwise DLI may support durable remission when used as a post-transplant maintenance strategy in chemo-refractory AML with adverse cytogenetics, such as t(1;3)(p36.3;q21). This maintenance approach warrants further investigation to clarify feasibility and safety.

## Introduction

Relapse remains the primary cause of treatment failure after allogeneic hematopoietic stem cell transplantation (allo-HSCT) for acute myeloid leukemia (AML), particularly in genetically high-risk patients (1, 2). One such adverse-risk abnormality is the translocation t(1;3)(p36.3;q21), a distinct chromosomal aberration seen in myeloid neoplasms, including AML (3, 4). In the International Consensus Classification, this entity is categorized as AML with other rare recurring translocations (5). Small case series have demonstrated a chemo-refractory disease course and poor long-term outcomes (6, 7), supporting upfront allo-HSCT in eligible patients. Still, post-transplant relapse remains a concern, underscoring the need for effective maintenance strategies in this high-risk AML subset.

Prophylactic donor lymphocyte infusion (DLI) may reduce post-transplant relapse in high-risk AML, although its efficacy has not been fully elucidated (8, 9). Given disease heterogeneity and the potential for immune escape, incorporating non-conventional cytoreductive approaches into DLI-maintenance may represent a rational strategy. However, optimal partner therapies and treatment schedules for DLI in the maintenance setting have not been defined.

Gemtuzumab ozogamicin (GO), a CD33-targeted antibody-drug conjugate, has demonstrated clinical efficacy in CD33-positive AML (10, 11). Recently, our group evaluated azacitidine (AZA) plus GO for post-transplant maintenance in high-risk AML showing that prophylactic administration of GO after allo-HSCT was feasible, although relapse remained frequent (12). While GO plus DLI has been described as salvage therapy for post-transplant relapse (13–15), its role as planned prophylactic maintenance after allo-HSCT remains undefined.

Here, we present a patient with chemo-refractory t (1,3)(p36.3;q21)-positive AML that relapsed early after a first allo-HSCT. The patient achieved long-term complete remission (CR) after a second, haploidentical allo-HSCT followed by low-dose GO and dose-escalated DLI maintenance. This case illustrates an immune-directed post-transplant maintenance approach using sequential low-dose GO followed by stepwise DLI, rather than salvage therapy, in a very high-risk AML setting.

## Case report

A 29-year-old woman was diagnosed with AML with myelodysplasia-related changes. A flow cytometric analysis showed that the blasts were positive for CD34, CD13, CD33, CD38, CD56, CD117, and HLA-DR and negative for CD14, CD41, CD10, CD19, CD3, CD7 and TdT. A chromosomal analysis by G-banding revealed 46, XX, t(1;3)(p36.3;q21). Targeted sequencing of bone marrow, performed as previously described (16), identified mutations in *GATA2* (variant allele frequency (VAF), 47.68%), *SF3B1* (VAF, 46.22%), *NRAS* (VAF, 42.84%), and *PTPN11* (VAF, 4.35%) (**Table 1**). Therefore, she was classified as having an adverse cytogenetic risk (17). She received two cycles of anthracycline-containing induction therapy but failed to achieve remission.

**Table 1:** Mutational status of representative genes.

Gene	CDS change	AA change	VAF at admission (%)	VAF at relapse (%)
*GATA2*	c.959G>A	p.Gly320Asp	47.68	10.25
*SF3B1*	c.860_863dupTCTG	p.Trp288CysfsTer12	46.22	9.72
*NRAS*	c.1734_1823dup	p.Val579_Glu608dup	42.84	8.87
*PTPN11*	c.1508G>T	p.Gly503Val	4.35	N.D

AA, amino acid; CDS, coding sequence; N.D, not detectable; VAF, variant allele frequency

**Figure 1:**
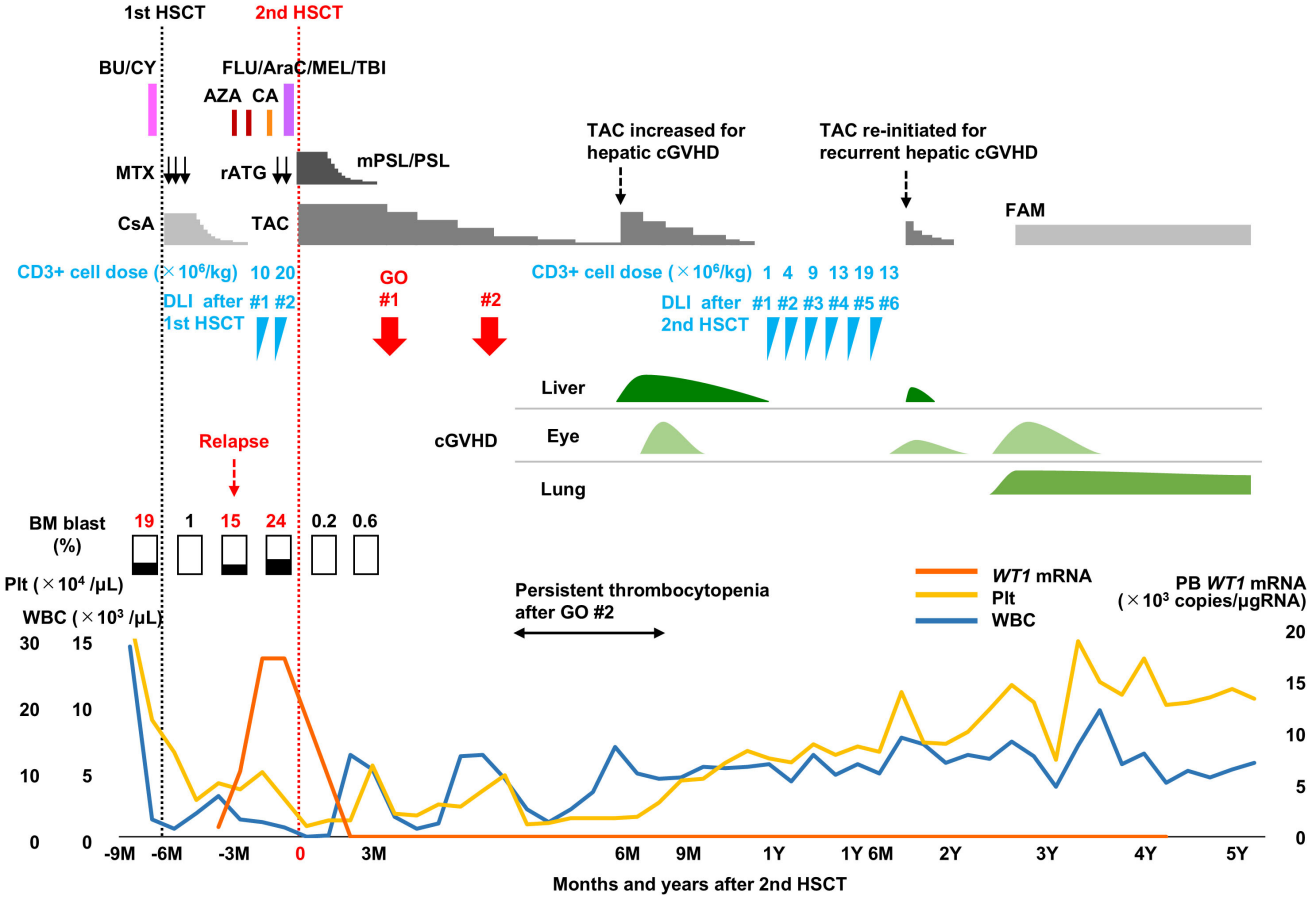
Clinical course. Treatment and cGVHD course, immunosuppressive therapy, WBC and Plt counts, PB *WT1* mRNA levels, and BM blast percentage are shown. Black and red dashed lines indicate the first and second allo-HSCT, respectively. Low-dose GO was administered on days 98 and 140 after the second allo-HSCT, followed by significant cytopenias and hepatic chronic GVHD onset requiring tacrolimus escalation on day 189. Ocular chronic GVHD also developed on day 224. Because additional GO administration was precluded by persistent grade 3 thrombocytopenia and two GO doses alone were considered insufficient as maintenance, DLI was selected as the next step in sequential maintenance. After hepatic and ocular chronic GVHD had stabilized and tacrolimus had been discontinued on day 339, DLI was initiated on day 374. AML, acute myeloid leukemia; AraC, cytarabine; rATG, rabbit antithymocyte globulin; AZA, azacitidine; BM, bone marrow; BU, busulfan; CA, aclarubicin + AraC; cGVHD, chronic graft-versus-host disease; CsA, cyclosporine; CY, cyclophosphamide; DLI, donor lymphocyte infusion; FAM, fluticasone propionate inhalation, azithromycin, and montelukast sodium; FLU, fludarabine; GO, gemtuzumab ozogamicin; HSCT, hematopoietic stem cell transplantation; MEL, melphalan; mPSL, methylprednisolone; MTX, methotrexate; PB, peripheral blood; Plt, platelet; PSL, prednisolone; TAC, tacrolimus; TBI, total-body irradiation.

Following referral to our hospital for allo-HSCT, MEC (mitoxantrone, etoposide, and cytarabine) was administered as salvage therapy; however, the disease remained refractory. Four months after diagnosis, she underwent bone marrow transplantation from her HLA-matched brother following conditioning with busulfan (12.8 mg/kg) and cyclophosphamide (120 mg/kg) (**Figure 1**). Graft-versus-host disease (GVHD) prophylaxis consisted of cyclosporine and short-term methotrexate. Neutrophil engraftment was achieved on day 17. On day 28, bone marrow examination through fluorescence *in situ* hybridization demonstrated CR with 0.8% blasts and full-donor chimerism. However, peripheral blood *WT1* mRNA was mildly elevated to 980 copies/μg RNA on day 53 and increased to 6,600 copies/μg RNA on day 82, in parallel with hematologic relapse characterized by 15.1% bone marrow blasts and mixed chimerism (XY: 84.2%, XX: 15.8%). Flow cytometry at relapse showed the same immunophenotype as at diagnosis, with blasts positive for CD34, CD13, CD33, CD38, CD56, CD117, and HLA-DR and negative for CD14, CD41, CD10, CD19, CD3, CD7, and TdT. G-banding and targeted sequencing (**Table 1**) also showed no significant changes from the initial diagnosis. Cyclosporine was tapered and discontinued by day 105. Despite two cycles of AZA and a first DLI on day 132 (CD3+ cell dose: 1.0 × 10^7^/kg), the patient remained refractory with persistent peripheral blood blasts. Salvage with CA (cytarabine and aclarubicin) on day 157 and a second DLI on day 160 (CD3+ cell dose: 2.0 × 10^7^/kg) failed to induce remission.

A second allo-HSCT from her HLA-haploidentical father was performed on day 189 after the first transplantation. The conditioning regimen consisted of fludarabine (180 mg/m^2^), cytarabine (8 g/m^2^), melphalan (140 mg/m^2^), total-body irradiation (4 Gy), and rabbit anti-human thymocyte globulin (2.5 mg/kg). GVHD prophylaxis consisted of tacrolimus and systemic corticosteroids, initially methylprednisolone, then oral prednisolone. Following neutrophil and platelet engraftment on days 12 and 22, she again achieved CR with full-donor chimerism on day 28 after the second allo-HSCT. Systemic corticosteroids were discontinued on day 77, and tacrolimus tapering was started on day 91. Given the extremely high risk of relapse, post-transplant maintenance therapy was initiated as soon as feasible after the second allo-HSCT. Two cycles of GO (3 mg/m^2^/dose) were administered on days 98 and 140 while the patient remained on low-dose tacrolimus. Subsequently, the patient developed grade 4 neutropenia and thrombocytopenia (CTCAE v5.0) without infectious or hemorrhagic complications. Total bilirubin remained within the normal range; however, grade 2 AST/ALT and grade 1 ALP/gamma-glutamyl transferase were observed. Although GO-related drug-induced liver injury was considered, the concurrent oral lichen planus-like changes supported a clinical diagnosis of hepatic chronic GVHD (NIH liver score 1), and tacrolimus was temporarily increased on day 189. Thrombocytopenia (grade 3) persisted until day 210, and no further GO was administered. Although ocular chronic GVHD (NIH eye score 2) also developed on day 224, both hepatic and ocular symptoms subsequently improved, allowing tacrolimus to be tapered and successfully discontinued by day 339. Because prolonged grade 3 thrombocytopenia had precluded additional GO administration, and two cycles of GO alone were considered insufficient as maintenance in this high-risk setting, DLI was selected as the next step in sequential maintenance. Its initiation was deferred until hepatic and ocular chronic GVHD had stabilized under tapering immunosuppression. After confirming the stability of GVHD, prophylactic DLI was initiated on day 374 and administered six times at 1-month intervals (CD3-positive cell dose: 1.0 × 10^6^ to 1.9 × 10^7^/kg). On day 626, hepatic chronic GVHD (NIH liver score 1) recurred, requiring re-initiation of tacrolimus. Following the normalization of liver function tests, tacrolimus was again successfully tapered and discontinued on day 767. Ocular chronic GVHD (NIH eye score 2) relapsed on day 940. Spirometry showed a forced expiratory volume in 1 second of 52.4% predicted, and chest computed tomography revealed air trapping consistent with bronchiolitis obliterans, leading to a diagnosis of pulmonary chronic GVHD (NIH lung score 2), corresponding to severe chronic GVHD by NIH global severity criteria. Therefore, FAM treatment (inhaled fluticasone propionate, azithromycin, and montelukast sodium) was initiated. Throughout the post-transplant course after the second allo-HSCT, peripheral blood *WT1* mRNA remained undetectable. The patient returned to work during the course of DLI therapy. Her chronic GVHD has remained clinically stable with a good Eastern Cooperative Oncology Group performance status of 1. At 6 years after the second allo-HSCT, she is in CR with full-donor chimerism. Longitudinal *WT1* mRNA monitoring and donor chimerism assessed by sex-mismatched fluorescence *in situ* hybridization are provided in **Supplementary Table 1**. Although *WT1* mRNA is an imperfect surrogate marker that may not always correspond directly to morphologic disease status, the combination of persistently undetectable *WT1* mRNA and sustained full-donor chimerism supported durable molecular remission in the present case.

## Discussion

A Japanese study reported that patients with AML relapsing within 3 months post-allo-HSCT show a dismal 2-year overall survival (OS) of 4%, with relapse being the primary cause of death (18). Even after a second allo-HSCT, the 2-year OS is 15%, with over 40% of patients relapsing again within one year (19). AML with t(1;3)(p36.3;q21) represents a particularly high-risk biological subset in which *RPN1* promoter-mediated *PRDM16* (*MEL1*) overexpression promotes stemness and impairs myeloid differentiation (3, 4, 20). *PRDM16* is a physiological regulator of hematopoietic stem cell maintenance, and its pathological overexpression in AML activates stem-cell-associated transcriptional programs, thereby promoting a stem-like phenotype that may contribute to resistance to standard chemotherapy. Although conceptually analogous to inv (3)/t(3;3), where a distal *GATA2* enhancer activates *MECOM* (*EVI1*), t(1;3)(p36.3;q21) constitutes a distinct form of 3q21-driven transcriptional deregulation (21). Clinically, in a review of 36 patients with t(1,3)(p36.3;q21), including 24 with *de novo* and secondary AML, only a minority achieved CR after chemotherapy, and the median OS was 21.3 months (22). Another study of seven patients reported a 5-year OS of 17.1% following intensive chemotherapy (6). These data suggest that conventional chemotherapy alone is insufficient, necessitating allogeneic immune-mediated graft-versus-leukemia (GVL) effects via allo-HSCT and, where appropriate, DLI. Consistent with this high-risk nature, our patient relapsed on day 82 after the first allo-HSCT and failed to achieve remission before the second allo-HSCT, providing a strong rationale for post-transplant maintenance.

Prophylactic or pre-emptive DLI enhances GVL effects through alloreactive T cells and has shown potential to improve long-term survival in high-risk AML (8, 9, 23). Several studies have investigated combining DLI with low-dose AZA; however, this strategy requires prolonged cyclic administration and is frequently complicated by myelosuppression, leading to treatment delays or discontinuation (24, 25). Moreover, most patients in previous cohorts were hypomethylating agent (HMA)-naive, and these results may not apply to HMA-refractory AML. Therefore, alternative agents to combine with DLI based on different mechanisms are warranted for high-risk, HMA-refractory cases.

Clinical data on GO as post-transplant maintenance are restricted to small series. We have previously demonstrated the feasibility of AZA (30 mg/m^2^ on days 1–7) followed by GO (3 mg/m^2^ on day 8) for up to four cycles; however, the 2-year relapse rate was 50% (12). Furthermore, grade 4 neutropenia and thrombocytopenia in nearly all patients, alongside 53.6% febrile neutropenia, hindered treatment continuity. Therefore, optimizing both safety and long-term efficacy remains a challenge for GO-based maintenance.

In our case, cytotoxic chemotherapy plus DLI after the first allo-HSCT failed to achieve remission, implying that DLI alone would be unlikely to control this highly refractory setting. Moreover, the patient showed AZA resistance, and venetoclax (VEN) was unavailable in Japan outside of clinical trials at that time, making AZA/VEN-based maintenance neither biologically nor clinically feasible. The absence of actionable driver mutations, such as *FLT3* or *IDH1/2* alterations, also limited molecularly targeted options. Hence, we selected a CD33-directed approach with low-dose GO. After two GO doses caused cumulative cytopenia, we continued with serial DLI to sustain GVL effects while limiting further toxicity.

To date, only three single-patient reports have described GO plus DLI after allo-HSCT, all used as salvage in the relapse setting rather than maintenance, as summarized in **Table 2** (13–15). Owing to the relapse settings, GO doses in these previous cases were higher than those in the present case. When GO was given for early relapse within 7 months post-transplant, profound cytopenia complicated by infectious or hemorrhagic events occurred, and chronic GVHD after DLI required systemic steroids, possibly reflecting high initial CD3-positive cell doses (13, 15). In contrast, in a case treated more than 1 year after allo-HSCT, neither major GO toxicity nor GVHD after DLI was observed, although the CD3-positive cell dose was not reported (14). Overall, the optimal integration of GO plus DLI, specifically regarding GO dosage, timing, and DLI escalation, remains to be defined. Comparative assessments of efficacy and toxicity should also be approached with caution, because the available reports are limited to single cases and lack systematic information on immune status.

**Table 2:** Previous case reports of patients treated with GO and DLI after allogeneic HSCT.

Reference	Age at HSCT/sex/ disease/ karyotype	Donor type/ graft source/ conditioning/ GVHD prophylaxis	Disease status and treatment intent at GO+DLI	GO (dose and timing after HSCT)	DLI (CD3+cell dose and timing after HSCT)	Response and duration	Hepatic AEs after GO administration	Other AEs after GO administration	cGVHD and treatment
Balduzzi, et al. (13)	2/female/ ALL/ t(4;11)	HLA-matched URD/BM/ BU+CY+VP-16/CsA+MTX+ATG	hRL; salvage	5 mg/m^2^ at 7 months and 2 mg/m^2^ at 7.5 months	5×10^6^/kg at 8 and 10 months, respectively	hCR, at least 10 months	None	Severe pancytopenia (unknown grade)	Skin/GI/liver; steroid+ATG
Tamai, et al. (14)	38/female/ AML/ t(6;11)	HLA-matched sibling/BM/ CY 60 mg/kg+ TBI 12 Gy/CsA+MTX	hRL: salvage	9 mg/m^2^ at 16 and 16.7 months, respectively	NA at 18 months	hCR, at least 8 months	No grade 3–4 hepatic toxicity or VOD/SOS	NA	None
Tachibana, et al. (15)	30/female/ AML/ inv(9)(p12q13)	HLA-matched sibling/PB/ CY 120 mg/kg+ TBI 12 Gy/ CsA+MTX	hRL; salvage	6 mg/m^2^ at 1.7 and 2.3 months, respectively	1×10^7^/kg at 2.6 months	hCR, at least 23 months	No VOD/SOS	Grade 4 neutropenia and thrombocytopenia, hemorrhagic pharyngitis, colitis, and cystitis	Eye/skin/ oral cavity; steroid
Present case	29/female/ AML/ t(1;3)	HLA-haploidentical father/BM/FLU 180 mg/m^2^+AraC 8 g/m^2^+MEL 140 mg/m^2^+TBI 4 Gy/ TAC+ATG+mPSL	mCR; prophylactic maintenance	3 mg/m^2^ at 3.3 and 4.7 months, respectively	1×10^6^–1.9×10^7^/kg, total of 6 times at 1-month intervals from 12.4 to 19.2 months	mCR, at least 60 months	Grade 2 AST/ALT elevation; no VOD/SOS.	Grade 4 neutropenia and thrombocytopenia	Eye/liver/lung; TAC +FAM treatment

AEs, adverse events; ALL, acute lymphoblastic leukemia; AML, acute myeloid leukemia; AraC, cytarabine; ATG, antithymocyte globulin; BM, bone marrow; cGVHD, chronic graft-versus-host disease; CsA, cyclosporine; CY, cyclophosphamide; DLI, donor lymphocyte infusion; FAM, fluticasone propionate inhalation, azithromycin, and montelukast sodium; FLU, fludarabine; GI, gastrointestinal; GO, gemtuzumab ozogamicin; hCR, complete remission; HSCT, hematopoietic stem cell transplantation; hRL, hematological relapse; mCR, molecular complete remission; MEL, melphalan; mPSL, methylprednisolone; PB, peripheral blood; TAC, tacrolimus; TBI, total-body irradiation; URD, unrelated donor; VOD/SOS, veno-occlusive disease/sinusoidal obstruction syndrome, VP-16, etoposide.

A key advantage of our GO-based strategy was its prophylactic use in minimal disease burden. Previously, salvage high-dose GO (9 mg/m^2^) combined with cytarabine for post-transplant AML relapse caused febrile neutropenia and grade 3–4 thrombocytopenia in all patients and grade 3–4 hepatic toxicity in 27% (26). Notably, 81% of those patients relapsed within 6 months after allo-HSCT, underscoring the safety concerns associated with intensive GO early after transplant. Other reports have similarly noted grade 3 hepatic injury or mild veno-occlusive disease/sinusoidal obstruction syndrome (VOD/SOS) after administering 8–9 mg/m^2^ GO beyond 100 days post-transplant (27, 28). However, non-transplant data suggest a low VOD/SOS risk when individual GO doses are 3 mg/m^2^ or less (29). Therefore, in a prophylactic context with minimal leukemic burden, low-dose GO at 3 mg/m^2^ may be biologically plausible, preserving antileukemic activity while potentially mitigating toxicity in the early post-transplant setting.

A prior experimental study reported that GO depletes CD33-positive myeloid-derived suppressor cells, which suppress T-cell activity (30). These findings provide a theoretical rationale that GO-mediated depletion of these suppressor cells might reduce T-cell suppression and create a more immune-favorable context for the GVL effects of subsequent DLI. However, no correlative immune analyses, such as assessment of myeloid-derived suppressor cells, T-cell subsets, or cytokines, were performed in this patient. Moreover, the interval between GO and DLI limits any inference regarding direct synergy. Thus, the immunomodulatory interaction between GO and DLI in this case remains hypothetical and requires future translational validation.

Following EBMT consensus recommendations, we administered stepwise, dose-escalated DLI (31). In haploidentical settings, higher CD3-positive cell doses were associated with moderate-to-severe GVHD and severe GVHD was linked to increased treatment-related mortality (32). In our 30-year institutional experience, however, DLI use after haploidentical transplantation has increased without elevating severe GVHD, coinciding with a decreasing trend in the initial CD3-positive cell dose (33). These findings suggest that low initial cell doses with stepwise escalation of DLI are a feasible and safe option for inducing GVL effects, even after haploidentical allo-HSCT. Our present case supports the potential clinical utility of this strategy and suggests that it may bridge the period before durable GVL immunity is fully established after transplantation.

In conclusion, this case suggests that sequential low-dose GO followed by stepwise DLI may support durable remission when used as a post-transplant maintenance strategy for chemo-refractory AML with adverse-risk cytogenetics, such as t (1,3)(p36.3;q21). However, the strategy was associated with severe cytopenias and GVHD-related complications, although these toxicities were ultimately manageable in this patient. This single case was limited by incomplete evaluation of prognostic indicators including long-term hematopoietic function and immune status. The relative contributions of GO and DLI, the optimal dosage and timing, and generalizability remain to be determined, underscoring the need for careful monitoring and further validation of this approach.

## Patient perspective

The patient developed AML at 29 years of age while actively working. Initially, she felt optimistic that the treatment would be effective. However, remission was not achieved, and the early relapse after the first allo-HSCT was profoundly discouraging. Following a second allo-HSCT, she was informed that the risk of relapse remained extremely high. Despite emotional distress, she elected to receive the maintenance therapy described in this report to reduce the likelihood of recurrence. She has since maintained long-term remission and has returned to full-time work as a hospital receptionist. Although she continues to experience dry eye due to ocular chronic GVHD, her dyspnea related to pulmonary chronic GVHD has improved, allowing her to resume normal daily activities. She expressed gratitude that the maintenance therapy was effective and hopes that further development of this approach will expand treatment options for patients with similarly high-risk disease.

## Data Availability

The original contributions presented in the study are included in the article/**Supplementary Material**. Further inquiries can be directed to the corresponding author.
