# Supplementary material for: Case Report: Immune-directed post-transplant maintenance with low-dose gemtuzumab ozogamicin and stepwise donor lymphocyte infusion in acute myeloid leukemia with t(1;3)(p36.3;q21)
Source: Front Immunol. 2026 Aug 5;17:1888993. doi: 10.3389/fimmu.2026.1888993 (PMC13485798; doi:10.3389/fimmu.2026.1888993)
Supplement: Supplementary file 1 [file SupplementaryFile1.docx]

| Supplementary Table S1. Longitudinal *WT1* mRNA levels and sex-mismatched FISH donor chimerism after the second allogeneic HSCT | | | | |
| --- | --- | --- | --- | --- |
| Days after  2nd-HSCT | Event | PB-*WT1* mRNA  (copies/µg RNA) | Chimerism  specimen | Chimerism  (%Donor) |
| -189 | 1st-HSCT |  |  |  |
| -162 |  |  | BM | 98.0 |
| -136 |  | 980 |  |  |
| -107 |  | 6600 | BM | 84.2 |
| -65 |  | 18000 |  |  |
| -37 |  | 18000 |  |  |
| 0 | 2nd-HSCT |  |  |  |
| 28 |  | <50 | BM | 100 |
| 56 |  | <50 |  |  |
| 98 | GO #1 | <50 | BM | 99.2 |
| 140 | GO #2 | <50 |  |  |
| 185 |  |  | PB | 99.6 |
| 210 |  |  | PB | 99.6 |
| 241 |  |  | PB | 99.0 |
| 283 |  |  | PB | 100 |
| 311 |  |  | PB | 99.6 |
| 339 |  |  | PB | 98.0 |
| 374 | DLI #1 | <50 | PB | 99.8 |
| 416 | DLI #2 | <50 | PB | 99.8 |
| 454 | DLI #3 | <50 | PB | 99.4 |
| 494 | DLI #4 | <50 | PB | 98.8 |
| 542 | DLI #5 | <50 | PB | 99.8 |
| 577 | DLI #6 |  | PB | 100 |
| 637 |  |  | PB | 100 |
| 675 |  |  | PB | 100 |
| 738 |  |  | PB | 99.6 |
| 794 |  |  | PB | 99.8 |
| 857 |  |  | PB | 100 |
| 948 |  |  | PB | 99.4 |
| 983 |  |  | PB | 99.6 |
| 1074 |  |  | PB | 99.8 |
| 1208 |  |  | PB | 99.8 |
| 1361 |  |  | PB | 100 |
| 1536 |  | <50 | PB | 99.4 |
| 1628 |  |  | PB | 99.8 |
| 1718 |  |  | PB | 99.6 |
| 1802 |  |  | PB | 99.8 |
| 1992 |  |  | PB | 99.0 |
|  |  |  |  |  |
| Abbreviations: PB, peripheral blood; BM, bone marrow; FISH, fluorescence in situ hybridization; HSCT, hematopoietic stem cell transplantation; GO, gemtuzumab ozogamicin; DLI, donor lymphocyte infusion. | | | | |
